# Integration of machine learning and mechanistic models accurately predicts variation in cell density of glioblastoma using multiparametric MRI

**DOI:** 10.1038/s41598-019-46296-4

**Published:** 2019-07-11

**Authors:** Nathan Gaw, Andrea Hawkins-Daarud, Leland S. Hu, Hyunsoo Yoon, Lujia Wang, Yanzhe Xu, Pamela R. Jackson, Kyle W. Singleton, Leslie C. Baxter, Jennifer Eschbacher, Ashlyn Gonzales, Ashley Nespodzany, Kris Smith, Peter Nakaji, J. Ross Mitchell, Teresa Wu, Kristin R. Swanson, Jing Li

**Affiliations:** 1 School of Computing, Informatics, and Decision Systems Engineering, Arizona State University, 699 S Mill Ave, Tempe, AZ 85281 USA; 2 Precision NeuroTherapeutics (PNT) Lab, Mayo Clinic Arizona, 5777 E Mayo Blvd, Phoenix, Arizona 85054 USA; 3 Department of Radiology, Mayo Clinic Arizona, 5777 E Mayo Blvd, Phoenix, Arizona 85054 USA; 4 Department of Pathology, Barrow Neurological Institute, Phoenix, Arizona USA; 5 Department of Neurosurgery, Barrow Neurological Institute, Phoenix, Arizona USA; 6 Department of Biostatistics and Bioinformatics, Moffitt Cancer Center, Tampa, Florida 33612 USA; 7 Department of Neurosurgery, Mayo Clinic Arizona, 5777 E Mayo Blvd, Phoenix, Arizona 85054 USA

**Keywords:** Cancer imaging, Scientific data

## Abstract

Glioblastoma (GBM) is a heterogeneous and lethal brain cancer. These tumors are followed using magnetic resonance imaging (MRI), which is unable to precisely identify tumor cell invasion, impairing effective surgery and radiation planning. We present a novel hybrid model, based on multiparametric intensities, which combines machine learning (ML) with a mechanistic model of tumor growth to provide spatially resolved tumor cell density predictions. The ML component is an imaging data-driven graph-based semi-supervised learning model and we use the Proliferation-Invasion (PI) mechanistic tumor growth model. We thus refer to the hybrid model as the ML-PI model. The hybrid model was trained using 82 image-localized biopsies from 18 primary GBM patients with pre-operative MRI using a leave-one-patient-out cross validation framework. A Relief algorithm was developed to quantify relative contributions from the data sources. The ML-PI model statistically significantly outperformed (p < 0.001) both individual models, ML and PI, achieving a mean absolute predicted error (MAPE) of 0.106 ± 0.125 versus 0.199 ± 0.186 (ML) and 0.227 ± 0.215 (PI), respectively. Associated Pearson correlation coefficients for ML-PI, ML, and PI were 0.838, 0.518, and 0.437, respectively. The Relief algorithm showed the PI model had the greatest contribution to the result, emphasizing the importance of the hybrid model in achieving the high accuracy.

## Introduction

Gadolinium contrast-enhanced T1-weighted (T1Gd) MRI serves as the clinical standard for guiding surgical resection and radiation therapy in glioblastoma (GBM), an aggressive primary brain tumor. While the contrast enhancement on a T1Gd MRI highlights the areas of a disrupted blood-brain-barrier and not the tumor cells directly^1^, this physiological disturbance does generally correlate with regions of higher tumor cell density^2^. Unfortunately, this rule-of-thumb correlation is not accurate enough to ensure that spatially targeted therapies, guided by the T1Gd, will provide optimal outcomes. More globally, this non-specificity impacts the ability to interpret how a given tumor responded to therapy, causing doubt in therapeutic decisions throughout the course of the disease^1,3,4^. Indeed, a wealth of evidence has shown that MRI enhancement underestimates true tumor burden, due to poor delineation of invasive tumor regions with intact BBB^5–8^, and that resection (better able to target the bulk of the tumor) results in an increase in overall survival^9^. These non-enhancing tumor populations can represent a substantial proportion of overall disease burden^7,9^, yet typically remain unresected (by surgery) and undertreated (by radiation), thereby leading to universal recurrence and poor survival.

There have been a number of attempts to use other types of clinical imaging to directly show the tumor invasion. Perhaps the most common is the Apparent Diffusion Coefficient (ADC) map, generated from diffusion MRI, which has previously been shown to inversely correlate with cell density^10–12^. However, this relationship to overall cell density is not easily converted to tumor cell density only, which is the primary variable of interest in the invasive rim of the tumor. Unfortunately, this uncertain area is the main region of interest for expanding spatially targeted therapies. Radio-labeled tracers used with positron emission tomography (PET) can also highlight actively proliferating regions within the brain^13–15^. While promising, the resolution of PET scans are not nearly as accurate for surgical planning as MRI and these scans are also more difficult to obtain due to the use of non-standard tracers with shorter half-lives and the need of a PET scanner^16^. In this paper, we present a hybrid method, consisting of machine learning and mechanistic model components, for predicting specifically the tumor cell density based on multi-parametric MRI.

Machine learning (ML) approaches have been gaining popularity in trying to elucidate tumor characteristics using radiological features^17–22^. Due to the limited data regarding spatial localization of tissue specimens (biopsies) from tumors typically collected, previous radiomics models have primarily aimed at categorizing the entirety of a tumor according to a given label derived from a single tissue sample, i.e. IDH1mut status, MGMT methylation status, or Verhaak tumor type^17,18,23^. We have made use of a unique data set consisting of multiple image-localized biopsies from 18 patients to enable spatially heterogeneous predictions of tumor cell density throughout an individual tumor.

Our approach is further unique in that it brings together the strengths of both machine learning (ML) and mechanistic models (MM). ML is very powerful in that it lets the data completely determine the relationship between the medical images and the variable being predicted. However, this can also be a weakness in that if there is not enough data to fully capture the uncertainties in a given relationship, ML is very susceptible to overfitting. MM can help fill in these gaps by encapsulating known relationships and feeding this supplemental knowledge to the ML model. For this purpose, we make use of the Proliferation-Invasion (PI) model of glioma growth, a model that can be made patient-specific and has been shown to have significance in predicting radiation sensitivity, benefit from resection, therapeutic response, and overall survival^24–29^. We thus refer to our hybrid model as the ML-PI model.

Following this introduction, we provide a brief background on the ML algorithms used and the PI model. We then present our methods including a succinct overview of how we combine the ML and PI models to form a single hybrid model. We then show the accuracy of our hybrid model is better than when compared to predictions from ML or PI alone. We also determine the significant contributing features to the ML-PI model demonstrating that the hybrid approach is critical to the success of this model.

## Methods

### Patient recruitment

Patients were recruited with clinically suspected GBM undergoing preoperative stereotactic MRI for first-line surgical resection prior to any treatment, as per the institutional review board approved protocol “Improving Diagnostic Accuracy in Brain Patients Using Perfusion MRI” at Barrow Neurological Institute (BNI). All patients provided written, informed consent prior to enrollment following the protocol procedures approved by BNI’s IRB. Data was collected and all protocol procedures were carried out in accordance with relevant guidelines and regulations. The patient cohort presented here has also been described in previous studies^19,20^. 82 biopsy samples were collected from 18 GBM patients, with each patient having 2–14 biopsy samples.

### Surgical biopsy

Pre-operative conventional MRI, including T1-Weighted gadolinium contrast-enhanced (T1Gd) and T2-Weighted sequences (T2W), was used to guide biopsy selection. Each neurosurgeon collected an average of 5–6 tissue specimens from each tumor by using stereotactic surgical localization, following the smallest possible diameter craniotomies to minimize brain shift. Specimens were collected from both enhancing mass (as seen on T1Gd) and non-enhancing brain around tumor (BAT), as seen on T2W, for each tumor. The neurosurgeons recorded biopsy locations via screen capture to allow subsequent coregistration with multiparametric MRI datasets. The biopsy tissue specimens were reviewed blinded to diagnosis by a neuropathologist and assessed for tumor content. Taking into account all visible cells (neurons, inflammatory cells, reactive glia, tumor cells, etc.), the percent tumor nuclei were estimated by a board-certified neuropathologist. Additional details of methods for surgical biopsy and pathological density measurement can be found in^19^.

### Multiparametric MRI and ROI segmentation

We included six multiparametric images in the present study, including T1Gd, T2W, dynamic contrast enhancement (EPI + C), mean diffusivity (MD), fractional anisotropy (FA), and relative cerebral blood volume (rCBV) (detailed MRI protocols and image co-registration can be found in^19^ and the Supplementary Information). The T2W ROI, region encompassing the abnormality on both the T2W and T1Gd images, of each tumor was manually segmented by a board-certified neuroradiologist as the target region for predictions.

### PI density estimation

The proliferation-invasion (PI) model aims to capture the most basic understanding of what cancer is: cells that grow uncontrollably and invade surrounding tissue. Mathematically, the PI model is written as follows:

$$\mathop{\overbrace{\frac{{\rm{\partial }}c}{{\rm{\partial }}t}}}\limits^{\,Rate\,of\,Change\,of\,Cell\,Density\,}=\mathop{\overbrace{{\rm{\nabla }}\cdot (D(x){\rm{\nabla }}c)}}\limits^{\,Invasion\,of\,Cells\,into\,Nearby\,Tissue\,}+\mathop{\overbrace{\rho c(1-\frac{c}{K})}}\limits^{\,Proliferation\,of\,Cells\,}$$

where *c*(*x*, *t*) is the tumor cell density, *D*(*x*) is the net rate of diffusion taken to be piecewise constant with different values in gray and white matter, *ρ* is the net rate of proliferation and *K* is the cell carrying capacity. This model has been used to predict prognosis^30^, radiation sensitivity^27^, benefit from resection^9^, and IDH1 mutation status^31^. Additionally, this model was used to create untreated virtual controls for use in defining response metrics that are more prognostically significant than those currently in use^28,32^.

Using the T1Gd and T2W images of each patient as input, a *D* and *ρ* value were computed for each patient^33^; these values were then used to produce a voxel-wise density estimation co-registered to the other images through the algorithm outlined in^34^.

### Image feature computation

An 8 × 8 voxel box was placed at the location of co-registered images and the PI-density map that corresponds to each biopsy sample. The average gray-level intensity over the 64 voxels within the box was computed for each image sequence as was the average PI-predicted tumor cell density. An additional slice of the MRI sequences was chosen for each patient from which we acquired unlabeled samples. The slice was selected such that the cross-section included a balanced amount of enhancing mass and non-enhancing BAT. 8 × 8 voxel boxes were placed one pixel apart on the T2W ROI of the chosen slice, and the average gray-level intensity and PI-predicted tumor cell density were calculated for each of these unlabeled samples, analogous to the labeled samples.

### Data augmentation by synthetic biopsies

As tissue is primarily acquired from regions suspected to be tumorous, the labeled samples were biased towards higher tumor cell densities (shown in Fig S1(a)). To provide a balanced dataset for ML-PI model training, synthetic biopsies, taken from regions expected to be low in tumor cell density, were identified for each patient (if necessary) to be included among the labeled data points. A total of 39 synthetic biopsy samples were added with each patient having 0–6 samples. Synthetic biopsies were treated in the same manner as the original labeled data for model training purposes. It is important to note that the synthetic biopsies were only used in model training, and not in validation of the model performance.

### Hybrid model development

**Figure 1:**
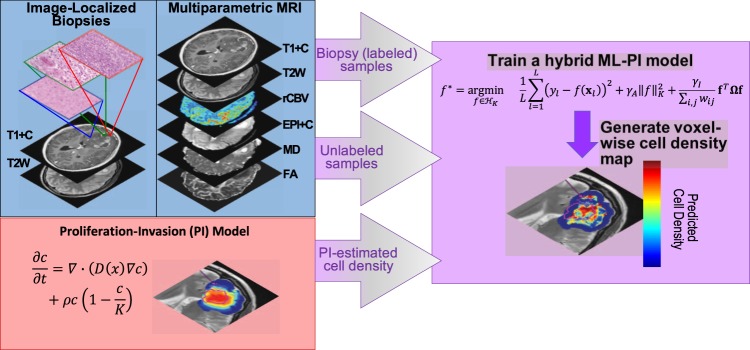
Workflow of building ML-PI and using the model to generate a predicted cell density map for the T2W ROI of each tumor/patient. Image-localized biopsies and multiparametric MRIs were collected for each patient in our study. The images were all co-registered and the voxel corresponding to the biopsy location was identified. *PI-model* Volumes of abnormality observed on T1Gd and T2 images were calculated via segmentation and were used to tune the PI model for each patient to provide a PI prediction of the tumor cell density. *Labeled Samples* For each image-localized biopsy, the mean intensity was calculated for each image sequence and PI cell density prediction corresponding to an 8 × 8 voxel window centered at the biopsy location. *Unlabeled Samples* A representative slice from the MRIs was selected. This slice was chosen such that it did not contain an image-localized biopsy location. The region of interest was segmented by a neuro-radiologist (LH) on this slice and a mean intensity from each MRI sequence and PI cell density was calculated from an 8 × 8 voxel window corresponding to every voxel contained within the region of interest. *Hybrid Model* These mean intensities from both labeled and unlabeled samples were used in the training of our hybrid ML-PI model. Validation tests were done using labeled sample data only.

Our hybrid model is comprised of two parts a semi-supervised learning approach and a mechanistic model. The general overview of the model is shown in Fig. 1.

#### Semi-supervised learning (SSL)

SSL has been widely used in applications in which labeled data are scarce but unlabeled data are available in large quantity, such as in our case in which there are very few biopsy-associated MRI voxels but numerous non-biopsy associated voxels. There are many types of SSL algorithms, but here we utilize a graph-based algorithm as presented in^35^ as our baseline model because of its proven high accuracy and efficiency in various applications, as well as its inductive learning ability that allows the trained model to be used to predict new patients. The core idea is to construct a graph with vertices being labeled and unlabeled samples in a training set and edges weighted by vertex proximity in the feature space.

#### Mechanistic Proliferation-Invasion (PI) model for patient-specific tumor cell density estimation

The vast majority of the clinically relevant PI literature, model discussed above, focuses on the intuition derived from the patient-specific parameter values (*D* and *ρ*), i.e. the gross tumor growth profile, rather than a voxel by voxel cell density prediction. This is exactly because the PI model smooths local regional cell density differences on this scale. The use of the PI model cell densities in the hybrid model presented here is for a similar purpose: these predictions provide an insight into the expected overall pattern but need to be augmented by more sophisticated data-driven ML methods to achieve local accuracy. That is, the biological insights provided by the PI model provide a means to regularize the ML models for tumor cell invasion.

#### Hybrid Model

The key concept in the creation of the hybrid ML-PI model is to incorporate PI-estimated regional cell density and imaging information from unbiopsied regions into a graph-based SSL. Briefly, it can be written mathematically as the solution to the following minimization problem:

1 $${f}^{\ast }={{\rm{argmin}}}_{f\in { {\mathcal H} }_{K}}(\mathop{\overbrace{\,\frac{1}{L}\sum _{l=1}^{L}\,{({y}_{l}-f({{\bf{x}}}_{l}))}^{2}}}\limits^{\,Prediction\,Error\,in\,Biopsied\,Samples\,}+\mathop{\overbrace{{\gamma }_{A}||f|{|}_{K}^{2}}}\limits^{Kernel\,\,Smoother}+\mathop{\overbrace{\frac{{\gamma }_{I}}{{\sum }_{i,j}{w}_{ij}}{{\bf{f}}}^{T}{\rm{\Omega }}{\bf{f}}}}\limits^{Integration\,of\,Unbiopsied\,Samples\,and\,PI}).$$

This model is an expansion from a typical supervised model, which would be composed of just the first two terms in eq. (1), by the third term incorporating the PI predictions and imaging information from unbiopsied regions. In this model, *L* is the number of biopsy samples in a training dataset. *y*_*l*_ is the pathologically measured tumor cell density for the *l*-th sample. **x**_*l*_ = (**z**_*l*_, *PI*_*l*_), where **z**_*l*_ contains gray-level intensity of each MRI sequence and *PI*_*l*_ is the PI predicted cell density (both **z**_*l*_ and *PI*_*l*_ contain values averaged over the 8 × 8 voxel box placed at the *l*-th biopsy sample location). *f*(**x**_*l*_) is a predictive function for cell density.$\,||\cdot |{|}_{K}^{2}$ is a norm on the reproducing kernel Hilbert space ${ {\mathcal H} }_{K}$. *γ*_*A*_ and *γ*_*l*_ are tuning parameters. **f** contains predictive density for each labeled and unlabeled sample, i.e., given *U* is the number of unlabeled samples, **f** = (*f*(**x**_1_), …, *f*(**x**_*L*_), *f*(**x**_*L*+1_), …, *f*(**x**_*L*+*U*_))^*T*^. **Ω** is a Laplacian matrix encoding the graph by holding the edge weight and connection information. Finally, *w*_*ij*_ are the edge weights between vertices *v*_*i*_ and *v*_*j*_, *i*, *j* = 1, …, *L* + *U*, of the graph capturing the relative difference in MRI features (*w*_*ij*,*z*_) and PI predictions (*w*_*ij*,*PI*_) can be computed using a product of two Gaussian functions, i.e.,

2 $${w}_{ij}={w}_{ij,z}\times {w}_{ij,PI}={\exp }(-\frac{||{{\bf{z}}}_{i}-{{\bf{z}}}_{j}||{}^{2}}{2{\psi }_{z}^{2}})\times {\exp }(-\frac{{(P{I}_{i}-P{I}_{j})}^{2}}{2{\psi }_{PI}^{2}}).$$

It can be shown, see Supplementary Information, that a solution to (1) exists and that it takes the form:

3 $${f}^{\ast }({\bf{x}})=\sum _{i=1}^{L+U}\,{\alpha }_{i}K({{\bf{x}}}_{i},{\bf{x}}),$$

for coefficients *α*_*i*_ and radial basis function kernel $K({{\bf{x}}}_{i},{{\bf{x}}}_{j})={e}^{-||{{\bf{x}}}_{i}-{{\bf{x}}}_{j}|{|}^{2}/2{\eta }^{2}}$ with tuning parameter *η* defining the width of the basis function. The *α*_*i*_’s can be determined analytically in terms of the three tuning parameters *γ*_*A*_, *γ*_*l*_, and *η*. This model and solution are discussed with much more detail and rigor in the Supplementary Material.

### Parameter Tuning

The hybrid ML-PI model includes three parameters that need to be tuned: *γ*_*A*_, which weights the kernel smoother, *γ*_*I*_, which weights the influence of unbiopsied regions and PI cell density prediction, and *η*, which is the width of the radial basis function kernel. The tuning ranges used were *γ*_*I*_, *γ*_*A*_ ∈ {10^−10^, …, 10^4^}; *η* ∈ {10^−1^, …, 10^2^}. We compared two tuning strategies: patient-specific tuning and uniform tuning. The former finds the optimal tuning parameters for each patient while the latter assumes the same optimal tuning parameters across all patients.

#### Patient-Specific Tuning

To individualize an ML-PI model for each patient, we utilized only the training samples from the other patients in the first term of Eq. (1). No real or synthetic biopsy samples from the target patient were used in training in order to avoid overfitting. Then, the trained model was used to predict the real biopsy samples of the target patient. The optimal tuning parameters were those that minimized the mean absolute prediction error (MAPE) of the real biopsies on the target patient. Selected parameters are given in Table S2.

#### Uniform Tuning

To find a single ML-PI model that could be applied to any patient, we looked for a single set of tuning parameters that minimized the MAPE across all patients. Thus, all patient samples were utilized in the first term of Eq. (1). Selected parameters are given in Table S3.

### Feature contribution analysis for ML-PI

To quantify the contribution of each feature, i.e. the mean image intensities and PI-estimated density, to the prediction made by ML-PI, we developed a modified Relief algorithm^36^, which we call “Relief-ML-PI”. This algorithm is run as a post-processing step and results in a score for each imaging feature *x*, *s*(*x*), that represents the contribution of *x*. Given *i* and *i*_*r*_ are samples in the training data set where *i*_*r*_ is the *r*^th^ nearest neighbor of *i* on the graph *G*, ${\hat{y}}_{i}$ and ${\hat{y}}_{{i}_{r}}$ are the predicted cell density of the two samples by ML-PI, ${\hat{y}}_{i}$ and ${\hat{y}}_{{i}_{r}}$ which correspond to feature measurements of *x*, *x*_*i*_ and ${x}_{{i}_{r}}$, we define *s*(*x*) as the difference between two probabilities, i.e.,

4 $$\begin{array}{rcl}s(x) & = & P({x}_{i}\,{\rm{and}}\,{x}_{{i}_{r}}are\,different|{\hat{y}}_{i}\,{\rm{and}}\,{\hat{y}}_{{i}_{r}}\,are\,different)\\  &  & -\,P({x}_{i}\,{\rm{and}}\,{x}_{{i}_{r}}are\,different|{\hat{y}}_{i}\,{\rm{and}}\,{\hat{y}}_{{i}_{r}}\,are\,similiar).\end{array}$$

The first term represents the probability that feature *x *is able to separate samples with different prediction values, while the second term represents the probability that *x* separates samples with similar prediction values. The larger the first probability and/or the smaller the second, the higher the *s*(*x*). Further discussion of this metric and our algorithm for computing the values is provided in the supplement.

## Results

### Difference in accuracy for different tuning strategies

In order to determine the degree to which patient difference would influence the optimal tuning parameters of ML-PI, we first compared the accuracy of ML-PI between the two training strategies, patient-specific and uniform. Table 1 shows the comparison result using two metrics: MAPE and Pearson correlation between the predicted and pathological cell density measurements. Both metrics allowed for a 5% error margin in the pathological measurement, i.e., if a predicted value is within ±5% of the pathological measurement, the prediction is considered correct (i.e., with zero prediction error). A MAPE of 0.106 means that if the pathologically measured density of a sample is *b*% (0 ≤ *b* ≤ 100), the predicted density by ML-PI deviates from *b*% by 10.6% on average. From Table 1 it is clear to see that patient-specific tuning has a significantly better accuracy than uniform tuning in terms of both a smaller MAPE (p < 0.0025) and a higher Pearson correlation (p < 0.001).

**Table 1:** Prediction accuracy of all models: Patient-specific ML-PI, Uniform ML-PI, PI only, and ML only.

	All Samples		BAT Samples Only	
	MAPE ± SD	Pearson Correlation	MAPE ± SD	Pearson Correlation
Patient-Specific ML-PI	0.106 ± 0.125	0.838	0.132 ± 0.118	0.820
Uniform ML-PI	0.176 ± 0.177	0.588	0.195 ± 0.179	0.504
PI	0.227 ± 0.215	0.437	0.204 ± 0.204	0.416
ML	0.199 ± 0.186	0.518	0.233 ± 0.209	0.208

Accuracy is considered for both cases of utilizing all samples or samples from the BAT only.

### Hybrid model more accurate than individual models

**Figure 2:**
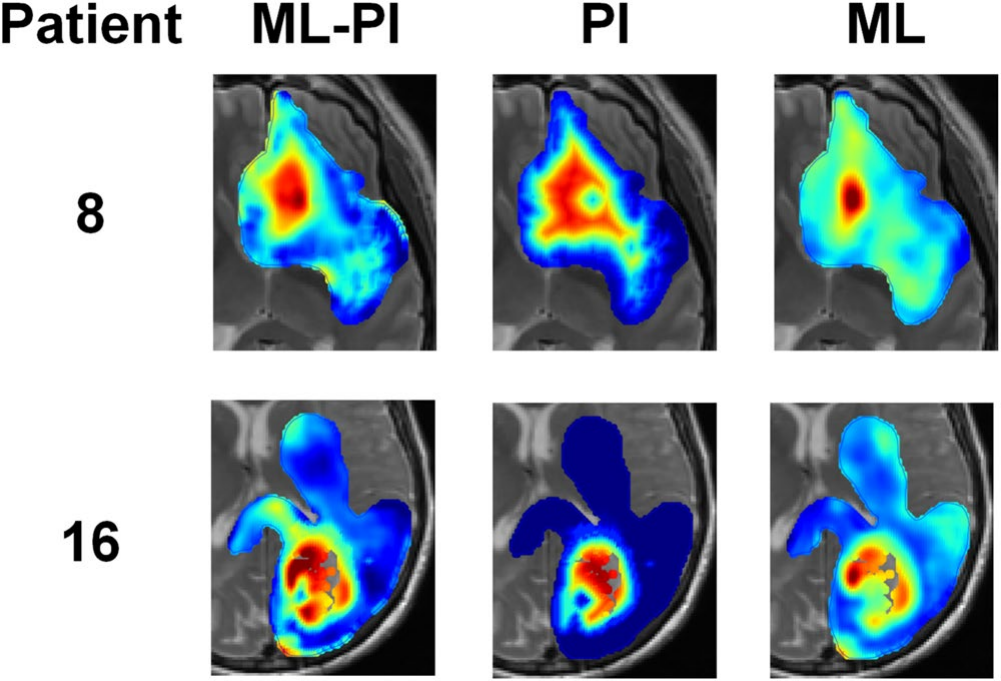
Illustrative spatial prediction maps resulting from three different models for two different patients. Red to blue colors represent 100–0% density. Models presented are the patient-specific hybrid ML-PI, the PI, and the ML. The weight given to the third term in Eq. (1) helps the hybrid model prediction to keep the general shape of the PI prediction and use information from unbiopsied regions, while the first term encourages accuracy in the prediction of biopsy samples and the second term promotes model stability/generalizability.

The output from each of these models is a spatially varying map of tumor cell density. Figure 2 provides illustrative examples of these for two patients. In Fig. 2, one can also see the effect on the prediction of combining the ML and PI models. It can be observed that the map by ML-PI generally conforms to the global shape of the PI map, but regional variations are allowed making its predictions more accurate than using PI and ML alone.

#### All Samples

**Figure 3:**
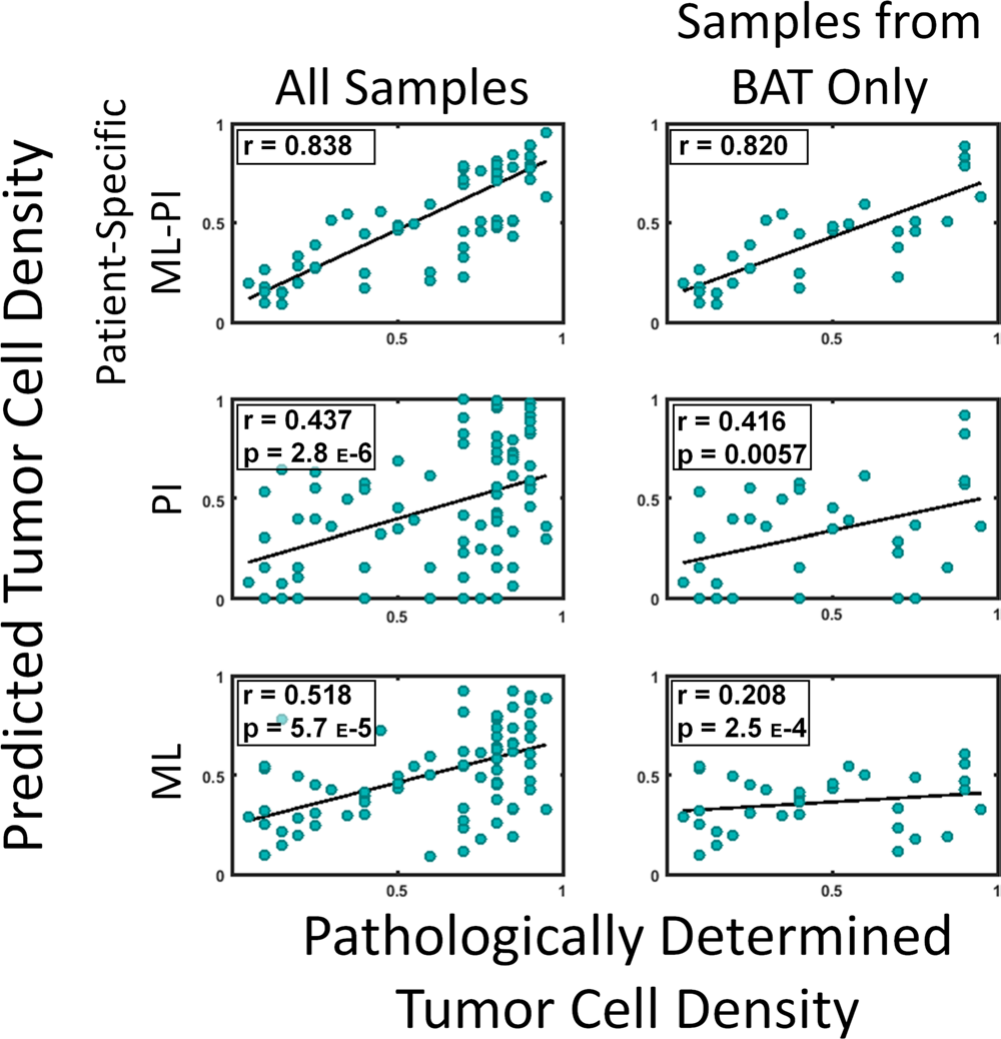
Prediction versus truth correlation plots. Here we show the scatter plots of prediction vs truth coming from three models, patient-specific ML-PI (top row), PI only (middle row) and ML only (bottom row). The plots in the left column include all 82 biopsy samples and the ML-PI and ML models were trained using all available samples. The plots in the right column include the prediction on only the 33 biopsy samples originating in the non-enhancing (BAT) region. The r value denotes the Pearson correlation coefficient. Correlation values for both columns are the highest for the patient-specific ML-PI model and significantly better than those corresponding to PI and ML alone (in both all samples and samples from BAT only). The p-values in the ML and PI plots correspond to comparing the model’s correlation to the correlation of the corresponding patient-specific ML-PI.

We wanted to test the performance of ML-PI against PI and ML used alone. The ML model is a supervised learning model that takes the same form of ML-PI except with *γ*_*I*_ = 0, i.e., a model that does not leverage unlabeled data. Table 1 shows the MAPE and Pearson correlation and Fig. 3 shows the plots of the predicted vs. pathological tumor cell density for each model. Compared with the patient-specific ML-PI, PI and ML alone had a significantly worse accuracy in terms of both MAPE and Pearson correlation (*p* < 0.001 in all comparisons). Also, we present the patient-wise MAPEs of ML-PI, PI, and ML in Table S1, found in the Supporting Information, to allow for comparison on the patient-level. ML-PI was able to predict more accurately than ML and PI in 17 out of 18 patients.

#### Brain Around Tumor (BAT) Samples Only

The BAT region is clinically interesting as that is most challenging to interpret through the obscured lens of MRI. Therefore, we further compared the performance of ML-PI, PI, and ML on predicting the tumor density of samples in the BAT. Out of the 82 total samples, 33 are in this area. The right two columns of Table 1 show the MAPE and Pearson correlation of each model and Fig. 3 additionally shows the predicted vs. pathological tumor cell density of the 33 samples for each of the three models. One can see from both Table 1 and Fig. 3 that ML-PI significantly outperforms PI and ML within the BAT region in all the comparisons (*p* < 0.05).

### Importance of Individual Tuning Parameters

We further investigated which of the three tuning parameters have a greater effect on model accuracy when allowed to be patient-specific. To achieve this purpose, we added a third tuning strategy, partially-uniform tuning, in which two of the three tuning parameters were kept the same across all patients while the remaining one was allowed to vary from patient to patient. This results in three models corresponding to *γ*_*A*_, *γ*_*I*_, or *η* as the parameter allowed to be patient-specific, respectively. Table 2 shows the performance of the three models. Compared with the result of uniform tuning in Table 1, we see patient-specific tuning of *γ*_*A*_ resulted in a significantly improved MAPE and Pearson correlation (*p* = 0.023 and 0.011). While patient-specific tuning of either *η *(*p* = 0.087 and 0.17)or *γ*_*I*_ (*p* = 0.22 and 0.35) alone does not result in a significantly improved MAPE and Pearson correlation. Selected tuning parameters are listed in Table S4.

**Table 2:** Prediction accuracy of ML-PI with partially-uniform tuning.

	Parameter allowed to be patient-specific		
	*γ*_*A*_	*γ*_*I*_	*η*
MAPE ± SD	0.127 ± 0.129	0.156 ± 0.154	0.140 ± 0.153
Pearson correlation	0.792	0.676	0.713

### Contributions from MRI sequences and PI

**Figure 4:**
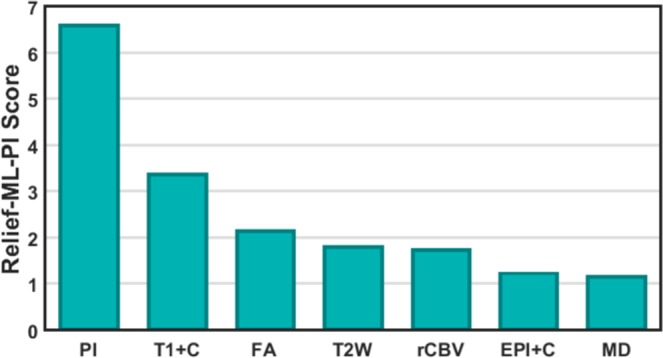
Contributions of PI and MRI sequences to ML-PI cell density prediction.

Using Relief-ML-PI, we can compute a contribution score for each image feature (one feature per MRI sequence) and PI from the ML-PI model specific to each patient. To identify the contributions aggregated over all the patients, we normalize the score of each feature within each patient to be between 0 and 1 by dividing the score by a sum over the scores of all the features. Then, the normalized scores from each patient are added together to produce an aggregated score showing contribution from each feature. Figure 4 shows the contribution from each MRI sequence and PI. It is clear that PI contributes the most.

## Conclusion

Despite the vast amounts of data being collected for individual patients, the promise of personalized medicine still remains to be fully realized. Data driven, machine learning processes provide a way to harness more and more of the incoming data. However, in the case of cancer, this avalanche of data still only represents limited snapshots of small regions of a complex, heterogeneous, and adapting system. Thus, machine learning models continue to be highly susceptible to overfitting and are notorious for not yielding reproducible results^37–39^. Mechanistic models can help guide machine learning models by providing information regarding the overarching principles guiding the system. This paper represents a key broad first step in demonstrating how combining these methodologies to achieve better results can be done.

In this paper, we have proposed a hybrid model leveraging principles from both machine learning and mechanistic models, the ML-PI model. This model aims to provide patient-specific spatial maps of tumor cell density within a given GBM patients’ brain for enhanced surgical and radiation therapy planning. This hybrid model leverages multiparametric MRI and PI tumor cell density predictions under a graph-based SSL framework. Both parameter tuning methods for the ML-PI model, patient-specific and uniform, were able to outperform the PI model alone and the ML model alone. Of note, the patient-specific tuning statistically significantly outperformed the other models, achieving a MAPE score of 0.1 and a correlation coefficient of 0.84 between predicted and true values. The importance of the hybrid paradigm was further emphasized through our Relief-ML-PI algorithm, which revealed that the PI tumor cell density prediction contributed most significantly to the prediction of all the included image features.

There are certainly limitations to the results presented in this paper. First and foremost, while the dataset is novel, it is small and only includes primary GBM patients. Thus, a leave-one-patient out framework was used as an initial validation check, but more data is certainly needed to supply a richer training and testing ground for this model and to explore the applicability of our model in the recurrent setting. Second, our model depends on six distinct MRI sequences, which are not all standardly acquired and some of which, e.g. perfusion, are known to vary significantly from institute to institute. Thus, the GBM patient population for which our model can be immediately utilized is limited. In future studies, we intend to study the decrease in accuracy as sequence requirements are removed in hopes of identifying a model that can be more broadly deployed. Third, the best accuracy was achieved for the patient-specific ML-PI, which does require a patient to already have had a biopsy in order for the prediction to be made. This certainly limits its ability to aid in surgical planning, but does not impact its potential usefulness in sculpting the dose map for radiation therapy. Further, we note that while the uniform model was not as accurate, it still outperformed the PI and ML models alone and could provide an initial prediction for an active sampling method to guide surgeons during surgery.

It is well accepted that GBM tumor cells exist beyond the enhancement observed on T1Gd MRI, the main surgical target. Radiation dose plans, implemented after surgery, try to account for this with broad margins of high dose out 2 cm from the enhancement and lower doses up to 4 cm away. Unfortunately, GBMs uniformly recur and the location of recurrence is almost always within the field of high dose radiation treatment. We believe the continued failure of radiation therapy is a direct result of the fact that standard-of-care dose plans and shapes are driven to be simultaneously aggressive and conservative. They are aggressive in wide margins by the knowledge that the cells are, but then are conservative in dose delivered due to the uncertainty in their precise location and the desire to preserve normal brain. We believe that as our model continues to be refined and is hopefully shown to be reliable, it can be used to better inform dose plans to be precisely aggressive and precisely conservative.

We are continuing to accrue patients from multiple institutions to our study to increase our dataset both in size and diversity. Future work will encompass retraining and testing our models with these new datasets and investigating possible other critical information that may produce more accurate models such as patient sex or tumor location. Should our model prove reliable, it will help localize invasive tumor populations within the otherwise non-specific T2W regions to better inform resection and radiation therapy, potentially increasing overall survival for GBM patients.

## Supplementary information

Supplementary Information

## Data Availability

The datasets generated during and/or analyzed during the current study are not publicly available due to institutional review board requirements but are available from the corresponding author on reasonable request.

## References

[1] Yang D (2016). Standardized MRI assessment of high-grade glioma response: A review of the essential elements and pitfalls of the RANO criteria. Neuro-Oncology Pract..

[2] Barajas RF (2010). Glioblastoma Multiforme Regional Genetic and Cellular Expression Patterns: Influence on Anatomic and Physiologic MR Imaging. Radiology.

[3] Quant EC, Wen PY (2011). Response assessment in neuro-oncology. Curr. Oncol. Rep..

[4] Okada H (2015). Immunotherapy response assessment in neuro-oncology: A report of the RANO working group. The Lancet Oncology.

[5] Silbergeld, D. L. & Chicoine, M. R. Isolation and characterization of human malignant glioma cells from histologically normal brain. *J. Neurosurg* (1997). 10.3171/jns.1997.86.3.0525 9046311

[6] Giese, A., Bjerkvig, R., Berens, M. E. & Westphal, M. Cost of migration: Invasion of malignant gliomas and implications for treatment. *Journal of Clinical Oncology* (2003). 10.1200/JCO.2003.05.063 12697889

[7] Schucht, P. *et al*. 5-ALA complete resections go beyond MR contrast enhancement: Shift corrected volumetric analysis of the extent of resection in surgery for glioblastoma. In *Acta Neurochirurgica* (2014). 10.1007/s00701-013-1906-7 24449075

[8] Gill BJ (2014). MRI-localized biopsies reveal subtype-specific differences in molecular and cellular composition at the margins of glioblastoma. Proc. Natl. Acad. Sci..

[9] Baldock, A. *et al*. Patient-specific Metrics of Invasiveness Reveal Significant Prognostic Benefit of Resection in a Predictable Subset of Gliomas. *PLoS One*, **9**(10) (2014). 10.1371/journal.pone.0099057 PMC4211670 25350742

[10] Sadeghi, N. *et al*. Apparent diffusion coefficient and cerebral blood volume in brain gliomas: Relation to tumor cell density and tumor microvessel density based on stereotactic biopsies. *Am. J. Neuroradiol* (2008). 10.3174/ajnr.A0851 PMC8118877 18079184

[11] Gupta, R. K. *et al*. Relationships between choline magnetic resonance spectroscopy, apparent diffusion coefficient and quantitative histopathology in human glioma. *J. Neurooncol* (2000). 10.1023/a:1006431120031 11263501

[12] Ellingson, B. M. *et al*. Spatially quantifying microscopic tumor invasion and proliferation using a voxel-wise solution to a glioma growth model and serial diffusion MRI. *Magn. Reson. Med* (2011). 10.1002/mrm.22688 PMC3065939 21413079

[13] Stockhammer, F., Plotkin, M., Amthauer, H., Landeghem, F. K. H. & Woiciechowsky, C. Correlation of F-18-fluoro-ethyl-tyrosin uptake with vascular and cell density in non-contrast-enhancing gliomas. *J. Neurooncol* (2008). 10.1007/s11060-008-9551-3 18317691

[14] Hutterer, M. *et al*. [18F]-fluoro-ethyl-l-tyrosine PET: A valuable diagnostic tool in neuro-oncology, but not all that glitters is glioma. *Neuro. Oncol* (2013). 10.1093/neuonc/nos300 PMC3578481 23335162

[15] Stockhammer, F. *et al* Association of F18-fluoro-ethyl-tyrosin uptake and 5-aminolevulinic acid-induced fluorescence in gliomas. *Acta Neurochir. (Wien)* (2009). 10.1007/s00701-009-0462-7 19639250

[16] Niyazi, M. *et al*. FET-PET for malignant glioma treatment planning. *Radiother. Oncol* (2011). 10.1016/j.radonc.2011.03.001 21458093

[17] Li, Z., Wang, Y., Yu, J., Guo, Y. & Cao, W. Deep Learning based Radiomics (DLR) and its usage in noninvasive IDH1 prediction for low grade glioma. *Sci. Rep*. (2017). 10.1038/s41598-017-05848-2 PMC5511238 28710497

[18] Bin Xi, Y. *et al*. Radiomics signature: A potential biomarker for the prediction of MGMT promoter methylation in glioblastoma. *J. Magn. Reson. Imaging* (2018). 10.1002/jmri.25860 28926163

[19] Hu, L. S. *et al*. Multi-parametric MRI and texture analysis to visualize spatial histologic heterogeneity and tumor extent in glioblastoma. *PLoS One*, **10**(11) (2015). 10.1371/journal.pone.0141506 PMC4658019 26599106

[20] Hu LS (2017). Radiogenomics to characterize regional genetic heterogeneity in glioblastoma. Neuro. Oncol..

[21] Korfiatis, P. *et al*. Comp-05. Evaluation of a Deep Learning Architecture for MRI Prediction of IDH, Ip19q and TERT in Glioma Patients. *Neuro. Oncol*. **20**(suppl_6), vi64 (2018).

[22] Prasanna P, Patel J, Partovi S, Madabhushi A, Tiwari P (2017). Radiomic features from the peritumoral brain parenchyma on treatment-naïve multi-parametric MR imaging predict long versus short-term survival in glioblastoma multiforme: Preliminary findings. Eur. Radiol..

[23] Yang, D., Rao, G., Martinez, J., Veeraraghavan, A. & Rao, A. Evaluation of tumor-derived MRI-texture features for discrimination of molecular subtypes and prediction of 12-month survival status in glioblastoma. *Med. Phys*. (2015). 10.1118/1.4934373 PMC5148162 26520762

[24] Harpold HLP, Alvord EC, Swanson KR (2007). The evolution of mathematical modeling of glioma proliferation and invasion. J. Neuropathol. Exp. Neurol..

[25] Swanson KR, Rostomily RC, Alvord EC (2008). A mathematical modelling tool for predicting survival of individual patients following resection of glioblastoma: a proof of principle. Br. J. Cancer.

[26] Szeto MD (2009). Quantitative metrics of net proliferation and invasion link biological aggressiveness assessed by MRI with hypoxia assessed by FMISO-PET in newly diagnosed glioblastomas. Cancer Res..

[27] Rockne R (2010). Predicting the efficacy of radiotherapy in individual glioblastoma patients *in vivo*: a mathematical modeling approach. Phys. Med. Biol..

[28] Neal, M. L. *et al*. Discriminating Survival Outcomes in Patients with Glioblastoma Using a Simulation-Based, Patient-Specific Response Metric. *PLoS One*, **8** (2013). 10.1371/journal.pone.0051951 PMC3553125 23372647

[29] Jackson, P. R., Juliano, J., Hawkins-Daarud, A., Rockne, R. C. & Swanson, K. R. Patient-Specific Mathematical Neuro-Oncology: Using a Simple Proliferation and Invasion Tumor Model to Inform Clinical Practice. *Bull. Math. Biol*. (2015). 10.1007/s11538-015-0067-7 PMC4445762 25795318

[30] Wang CH (2009). Prognostic significance of growth kinetics in newly diagnosed glioblastomas revealed by combining serial imaging with a novel biomathematical model. Cancer Res..

[31] Baldock AL (2014). Invasion and proliferation kinetics in enhancing gliomas predict IDH1 mutation status. Neuro. Oncol..

[32] Neal ML (2013). Response classification based on a minimal model of glioblastoma growth is prognostic for clinical outcomes and distinguishes progression from pseudoprogression. Cancer Res..

[33] Swanson, K. R., Alvord, E. C., Murray, J. D. & Rockne, R. C. Method and system for characterizing tumors. US8571844 B2 (2013).

[34] Konukoglu, E. *et al* A recursive anisotropic fast marching approach to reaction diffusion equation: application to tumor growth modeling. *Inf. Process. Med. Imaging* (2007). 10.1007/978-3-540-73273-0_57 17633740

[35] Belkin, M., Niyogi, P. & Sindhwani, V. Manifold regularization: A geometric framework for learning from labeled and unlabeled examples. *J. Mach. Learn. Res*. (2006).

[36] Robnik-Šikonja, M. & Kononenko, I. Theoretical and Empirical Analysis of ReliefF and RreliefF. *Mach. Learn*. (2003).

[37] Kumar, V. *et al*. Radiomics: The process and the challenges. *Magn. Reson. Imaging* (2012). 10.1016/j.mri.2012.06.010 PMC3563280 22898692

[38] Gillies, R. J., Kinahan, P. E. & Hricak, H. Radiomics: Images Are More than Pictures, They Are Data. *Radiology* (2016). 10.1148/radiol.2015151169 PMC4734157 26579733

[39] Limkin, E. J. *et al*. Promises and challenges for the implementation of computational medical imaging (radiomics) in oncology. *Annals of Oncology* (2017). 10.1093/annonc/mdx034 28168275

