# Supplementary material for: Integration of machine learning and mechanistic models accurately predicts variation in cell density of glioblastoma using multiparametric MRI
Source: Sci Rep. 2019 Jul 11;9:10063. doi: 10.1038/s41598-019-46296-4 (PMC6624304; doi:10.1038/s41598-019-46296-4)
Supplement: Supplementary file 1 — Supplementary Information [file 41598_2019_46296_MOESM1_ESM.docx]

**Supplementary information for**

**Integration of machine learning and mechanistic models accurately predicts variation in cell density of glioblastoma using multiparametric MRI**

Nathan Gaw^1*^, Andrea Hawkins-Daarud^2*^, Leland S. Hu^3^, Hyunsoo Yoon^1^, Lujia Wang^1^, Yanzhe Xu^1^, Pamela R. Jackson^2^, Kyle Singleton^2^, Leslie C. Baxter^3^, Jennifer Eschbacher^4^, Ashlyn Gonzales^3^, Ashley Nespodzany^3^, Kris Smith^5^, Peter Nakaji^5^, J. Ross Mitchell^6^, Teresa Wu^1^, Kristin Swanson^2,7*^, and Jing Li^1*^

^1^School of Computing, Informatics, and Decision Systems Engineering, Arizona State University, 699 S Mill Ave, Tempe, AZ 85281 USA.

^2^Precision NeuroTherapeutics (PNT) Lab, Mayo Clinic Arizona, 5777 E Mayo Blvd, Phoenix, Arizona 85054 USA.

^3^Department of Radiology, Mayo Clinic Arizona, 5777 E Mayo Blvd, Phoenix, Arizona 85054 USA.

^4^Department of Pathology, Barrow Neurological Institute, Phoenix, Arizona,

^5^Department of Neurosurgery, Barrow Neurological Institute, Phoenix, Arizona

^6^Department of Biostatistics and Bioinformatics, Moffitt Cancer Center, Tampa, Florida, 33612, USA

^7^Department of Neurosurgery, Mayo Clinic Arizona, 5777 E Mayo Blvd, Phoenix, Arizona 85054 USA.

*Contributed equally

**Supplementary Methods**

**Development of a hybrid ML-PI model**

The basic idea of ML-PI is to incorporate PI-estimated regional cell density and imaging information from unbiopsied regions into a graph-based SSL. ML-PI is a significant expansion from a typical supervised model that takes the following form:

$f^{*}=\underset{f\in\mathcal{H}_{K}}{\mathrm{argmin}} \frac{1}{L}\sum_{l=1}^{L} {(y_{l}-f\left( \mathbf{z}_{l} \right))}^{2}+\gamma_{A}\left\| f \right\|_{K}^{2}$. (1)

$L$ is the number of biopsy samples in a training dataset. $y_{l}$ is the pathologically measured tumor cell density for the $l$-th sample. $\mathbf{z}_{l}$ contains gray-level intensity of each MRI sequence averaged over the 8x8 voxel box placed at the $l$-th biopsy sample location. $f\left( \mathbf{z}_{l} \right)$ is a predictive function for cell density. ${(y_{l}-f\left( \mathbf{z}_{l} \right))}^{2}$ is a loss function that measures the discrepancy between the pathological and predicted density of each biopsy sample. $f$ is a function on the reproducing kernel Hilbert space (RKHS), $\mathcal{H}_{K}$, with a Mercer kernel $K$. $\left\| f \right\|_{K}^{2}$ is a norm on $\mathcal{H}_{K}$, which encourages stability and generalizability of the solution. $\gamma_{A}$ is a tuning parameter.

Equation (1) is a supervised learning model because it uses only the biopsy samples (labeled data). To incorporate unlabeled data and PI-estimated density into the model, we follow the idea of SSL and build a graph on all labeled and unlabeled samples. Specifically, one graph $G=\left( \mathbf{V},\mathbf{W} \right)$ is built for each patient. $\mathbf{V}$ is the set of vertices and $\mathbf{W}$ contains the weight of edge between each pair of vertices. Let $n=L+U$ be the number of vertices of the graph. $L$ is the number of all biopsy samples and $U$ is the number of voxels on the T2W ROI for the target patient. The edge weight between vertices $v_{i}$ and $v_{j}$, $i,j=1,\ldots,n$, can be computed using a product of two Gaussian functions, i.e.,

$w_{ij}\boldsymbol{=}w_{ij,z}\boldsymbol{\times}w_{ij,PI}\boldsymbol{=}exp\left( -\frac{\left\| \mathbf{z}_{i}\mathbf{-}\mathbf{z}_{j} \right\|^{2}}{2\psi_{z}^{2}} \right)\boldsymbol{\times}exp\left( -\frac{\left( {PI}_{i}\mathbf{-}{PI}_{j} \right)^{2}}{2\psi_{PI}^{2}} \right)$. (2)

${PI}_{i}$ is PI-estimated cell density averaged over the 8x8 box centered at the $i$-th voxel. $\mathbf{z}_{i}$ contains gray-level intensity of each MRI sequence averaged over the 8x8 box centered at the $i$-th voxel.

In essence, $w_{ij}$ reflects the closeness between two samples/vertices in terms of their respective image features ($w_{ij,z}$**)** and PI estimations ($w_{ij,PI}\boldsymbol{)}$. $\psi_{z}$ and $\psi_{PI}$ are parameters to adjust contributions to the weight from image features and PI, respectively.

Furthermore, the graph $G$ can be encoded into a Laplacian matrix defined as $\boldsymbol{\Omega=D-W}$, where $\mathbf{D}$ is the vertex degree matrix, i.e., a diagonal matrix with diagonal elements being the total sum of edge weights associated with each vertex, and $\mathbf{W}$ is the matrix of all the edge weights. Then, the model in (1) can be augmented by incorporating the graph Laplacian matrix, which gives the proposed ML-PI model as:

$f^{*}=\underset{f\in\mathcal{H}_{K}}{\mathrm{argmin}} \frac{1}{L}\sum_{l=1}^{L} \left( y_{l}-f\left( \mathbf{x}_{l} \right) \right)^{2}+\gamma_{A}\left\| f \right\|_{K}^{2}+\frac{\gamma_{I}}{\sum_{i,j} w_{ij}}\mathbf{f}^{T}\boldsymbol{\Omega f}$. (3)

$\mathbf{x}_{l}\boldsymbol{=}\left( \mathbf{z}_{l},{PI}_{l} \right)$. $\mathbf{f}$ contains predictive density for each labeled and unlabeled sample, i.e., $\mathbf{f}=\left( f\left( \mathbf{x}_{1} \right),\ldots,f\left( \mathbf{x}_{L} \right),f\left( \mathbf{x}_{L+1} \right),\ldots,f\left( \mathbf{x}_{L+U} \right) \right)^{T}$. $\sum_{i,j} w_{ij}$ is a sum of all the edge weights in the graph. Because of patient heterogeneity, we found that the graph of each patient has a wide range of sparsity levels, which causes difficulty in choosing a common search range for the tuning parameter $\gamma_{I}$. Adding $\sum_{i,j} w_{ij}$ solves this problem by normalizing patient-specific graphs to allow for $\gamma_{I}$ to be tuned within a common range.

Through some algebra, the last term in (3) can be shown to become:

$\mathbf{f}^{T}\boldsymbol{\Omega f=}\sum_{i,j=1}^{L+U} {(f\left( \mathbf{x}_{i} \right)-f(\mathbf{x}_{j}))}^{2}w_{ij,z}\boldsymbol{\times}w_{ij,PI}$. (4)

Then, it is clear that the minimization in (3) pushes samples that are closer in image features (i.e., with a larger $w_{ij,z}$) and in PI estimations (i.e., with a larger $w_{ij,PI}$) to have more similar predictions. This is traded off with the loss on the labeled data (the first term in (3)) and the smoothness of the predictive function in RKHS (the second term in (3)). In the extreme case when $w_{ij,z}\boldsymbol{=}w_{ij,PI}\boldsymbol{=}0$ for all the edges, (3) becomes the supervised learning model in (1). In essence, the role of PI in the proposed model is to regularize the learning of the predictive function in order to make sure the spatial proximity of predicted densities conform with that of PI densities to some extent. This implicitly takes into account the bio-mechanism of tumor growth, which is the foundation of the PI model.

**Theorem 1:** The solution of the optimization in (3) is the following expansion in terms of both labeled and unlabeled samples:

$f^{*}\left( \mathbf{x} \right)=\sum_{i=1}^{L+U} \alpha_{i}K(\mathbf{x}_{i},\mathbf{x})$, (5)

where $\mathbf{x}$ is any sample for which the cell density is to be predicted, which can be an unlabeled sample included in the ML-PI model in (3) or not (e.g., a sample outside the ROI or on a different slice of the tumor). $\alpha_{i}$’s are coefficients.

**Proof of Theorem 1**

This proof relies on the Representer Theorem^1^. Given any function $f\in\mathcal{H}_{K}$, $f$ can be uniquely comprised of $f_{\parallel}$ and $f_{\perp}$, where $f_{\parallel}$ is in the linear subspace spanned by the kernel functions ${\{K\left( \mathbf{x}_{i},\cdot\right)\}}_{i=1}^{L+U}$ and $f_{\perp}$ is the orthogonal component. By the reproducing property, the value of $f$ on any point $\mathbf{x}_{j}, 1\leq j\leq L+U$ is independent of $f_{\perp}$, as shown below:

$$f\left( \mathbf{x}_{j} \right)=$$

$\left\langle f,K(\mathbf{x}_{j},\cdot) \right\rangle=\left\langle\sum_{i=1}^{L+U} \alpha_{i}K\left( \mathbf{x}_{i},\cdot\right),K(\mathbf{x}_{j},\cdot) \right\rangle+\left\langle f_{\perp},K(\mathbf{x}_{j},\cdot) \right\rangle$

It follows that $\left\langle K\left( \mathbf{x}_{i},\cdot\right),K(\mathbf{x}_{j},\cdot) \right\rangle=K\left( \mathbf{x}_{i},\mathbf{x}_{j} \right)$ and $\left\langle f_{\perp},K(\mathbf{x}_{j},\cdot) \right\rangle$ vanishes. Therefore, the above formulation simplifies to

$f\left( \mathbf{x}_{j} \right)= \sum_{i=1}^{L+U} \alpha_{i}K(\mathbf{x}_{i},\mathbf{x}_{j})$,

which means that the terms of the optimization in (3) only rely on the gram matrix of the kernel function and the coefficients ${\{\alpha_{i}\}}_{i=1}^{L+U}$. Furthermore, the norm of $f$ in $\mathcal{H}_{K}$ has the following decomposition:

$\left\| f \right\|_{K}^{2}=\left\| \sum_{i=1}^{L+U} \alpha_{i}K(\mathbf{x}_{i},\cdot) \right\|_{K}^{2}+\left\| f_{\perp} \right\|_{K}^{2}\geq\left\| \sum_{i=1}^{L+U} \alpha_{i}K(\mathbf{x}_{i},\cdot) \right\|_{K}^{2}$

The above inequality is true because $f_{\perp}$ will only increase $\left\| f \right\|_{K}^{2}$, so it follows that the minimizer of (3) must result in $f_{\perp}=0$, leading to

$f^{*}\left( \mathbf{x} \right)=\sum_{i=1}^{L+U} \alpha_{i}K(\mathbf{x}_{i},\mathbf{x})$. $∎$

**Feature contribution analysis for ML-PI**

It is important to determine the quantitative contribution of each feature (i.e., imaging features and PI-estimated density) to the prediction made by ML-PI. It is a reasonable belief that all of the included MRI sequences and PI are biologically relevant to tumor cell density. Therefore, inclusion of all of them as features in building the ML-PI model is valuable, while their relative contributions may vary. Thus, instead of employing feature selection (a step prior to building a predictive model with purpose of removing irrelevant features), we choose to use a post-processing step that identifies how much each feature contributes to the prediction.

Let $x$ be a feature used in ML-PI, which can be a feature computed from an MRI sequence or PI-estimated cell density. Our objective is to compute a score for $x$, $s\left( x \right)$, that represents the contribution of $x$. To achieve this, we develop an algorithm based on the well-known Relief algorithm^2^, which we call “Relief-ML-PI”. Note that Relief was developed as a feature selection algorithm for supervised learning models. Our innovation in this paper is to modify it to become a post-analysis algorithm for feature contribution analysis of SSL models, specifically the ML-PI model. The proposed definition of $s\left( x \right)$ is the following: let $\mathbf{T}$ be the training dataset from which ML-PI is built. $\mathbf{T}$ includes both labeled and unlabeled samples. Let $i$ and $i_{r}$ be samples in $\mathbf{T}$; $i_{r}$ is the $r$^th^ nearest neighbor of $i$ on the graph $G$. Furthermore, consider the predicted cell density of the two samples by ML-PI, $\hat{y}_{i}$ and $\hat{y}_{i_{r}}$, and their respective measurements on feature $x$, $x_{i}$ and $x_{i_{r}}$. The definition of $s\left( x \right)$ can be based on the difference between two probabilities, i.e.,

$s\left( x \right)$ = $P\left( \left. x_{i}\mathrm{and}x_{i_{r}}are different \right|\hat{y}_{i}\mathrm{and}\hat{y}_{i_{r}} are different \right)$

$-P\left( \left. x_{i}\mathrm{and}x_{i_{r}}are different \right|\hat{y}_{i}\mathrm{and}\hat{y}_{i_{r}} are similiar \right)$. (7)

The first term represents the probability that feature $x$is able to separate samples with different prediction values, while the second term represents the probability that $x$ separates samples with similar prediction values. The larger the first probability and the smaller the second, the higher the $s\left( x \right)$. Furthermore, using the Bayes’ rule, we can write (7) as:

$s\left( x \right)$=$\frac{P\left( \hat{y}_{i}\mathrm{and}\hat{y}_{i_{r}} are diff.|x_{i}\mathrm{and}x_{i_{r}}are diff. \right)\times P\left( x_{i}\mathrm{and}x_{i_{r}}are diff. \right)}{P\left( \hat{y}_{i}\mathrm{and}\hat{y}_{i_{r}} are diff. \right)}$

$-\frac{\left\{ 1-P\left( \hat{y}_{i}\mathrm{and}\hat{y}_{i_{r}} are diff.|x_{i}\mathrm{and}x_{i_{r}}are diff. \right) \right\}\times P\left( x_{i}\mathrm{and}x_{i_{r}}are diff. \right)}{1-P\left( \hat{y}_{i}\mathrm{and}\hat{y}_{i_{r}} are diff. \right)}$. (8)

The format of $s\left( x \right)$ in (8) makes it relatively easier than (7) to develop an algorithm to estimate $s\left( x \right)$. The algorithm, Relief-ML-PI, is presented in Algorithm 1. The basic idea is to randomly select $m$ samples from $\mathbf{T}$. For each $i=1,\ldots,m$, find its $k$ nearest neighbors $i_{r}$, $r=1,\ldots,k$. Then, estimate the probabilities in (8) and eventually the $s\left( x \right)$ using lines 7-9 of the algorithm, in which

$d\left( \hat{y}_{i}, \hat{y}_{i_{r}} \right)=\frac{\left| \hat{y}_{i}- \hat{y}_{i_{r}} \right|}{max\left( \hat{y}_{j}|j\in\mathbf{T} \right)-min\left( \hat{y}_{j}|j\in\mathbf{T} \right)}$,

$d\left( x_{i}, x_{i_{r}} \right)=\frac{\left| x_{i}- x_{i_{r}} \right|}{max\left( x_{j}|j\in\mathbf{T} \right)-min\left( x_{j}|j\in\mathbf{T} \right)}$,

as the normalized difference between the response variables or feature values of two samples, and

$\delta\left( i,i_{r} \right)= \frac{\delta^{'}(i,i_{r})}{\sum_{l=1}^{k} \delta^{'}(i,i_{r})}$, $\delta^{'}\left( i,i_{r} \right)=e^{-{(\frac{rank(i,i_{r})}{\sigma})}^{2}}$.

$\delta^{'}\left( i,i_{r} \right)$ weights each of the $k$ nearest neighbors for sample $i$ and $\delta\left( i,i_{r} \right)$ normalizes the weights. We choose to use the rank of the $k$ nearest neighbors instead of computing the numerical distance due to the same reason as Relief, i.e., to make sure different samples are equally accounted for.

| **Algorithm 1** Relief-ML-PI |
| --- |

**Input:** measurement data $x_{i}$ and predicted response $\hat{y}_{i}$ for each

sample in training set $\mathbf{T}$; tuning parameters $m$, $k$.

**Output:** $s\left( x \right)$

1. **Initialize:**
2. $s\left( x \right)\leftarrow0$; $N_{dy}\left( x \right)\leftarrow0$; $N_{dx}\left( x \right)\leftarrow0$; $N_{dy\&dx}\left( x \right)\leftarrow0$;
3. **for** $i=1$ **to** $m$ **do**
4. Randomly select a sample $i$ from $\mathbf{T}$;
5. Find $k$ nearest neighbors for sample $i$, $i_{1},\ldots,i_{k}$ on graph

$G$;

1. **for** $r=1$ **to** $k$ **do**
2. $N_{dy}\left( x \right)\leftarrow N_{dy}\left( x \right)+d\left( \hat{y}_{i}, \hat{y}_{i_{r}} \right)\times\delta\left( i,i_{r} \right)$;
3. $N_{dx}\left( x \right)\leftarrow N_{dx}\left( x \right)+d\left( x_{i}, x_{i_{r}} \right)\times\delta\left( i,i_{r} \right)$;
4. $N_{dy\&dx}\left( x \right)\leftarrow N_{dy\&dx}\left( x \right)+d\left( \hat{y}_{i}, \hat{y}_{i_{r}} \right)\times$

$d\left( x_{i}, x_{i_{r}} \right)\times\delta\left( i,i_{r} \right)$;

1. **end for**
2. **end for**
3. $s\left( x \right) \leftarrow\frac{N_{dy\&dx}\left( x \right)}{N_{dy}\left( x \right)}-\frac{N_{dx}\left( x \right)-N_{dy\&dx}\left( x \right)}{m-N_{dy}\left( x \right)}$;

**Virtual biopsy selection procedure**

*Step 1:* For each patient, count the number of biopsy samples with density >70%. Denote this number by $r$. $r’$is the number of real biopsies with low-density. $v =r-r’$ is the number of virtual biopsy samples with low-density (<30%) that are to be found, in order to create balanced samples for the patient.

*Step 2:* Locate the BAT for the patient by subtracting the ROI segmented on T1+C from the ROI segmented on T2W. On the PI-estimated density map over the BAT, pick a sub-area to take virtual biopsy from according to the following biological criteria:

1. The sub-area needs to be away from the skull and the midline of the brain, since PI estimation tends to be less accurate at locations with physical barriers.
2. The sub-area should be close to the peripheral of the T2W ROI, where there is much lower chance to harbor high cell density.
3. Considering spatial continuity of cell density distribution, the PI estimation at a neighborhood of the biopsy sample should be more likely to be accurate if there is a real biopsy sample with low density whose PI density is also low. If the density of the real biopsy sample disagrees with PI density, the neighborhood of the sample should be avoided.

*Step 3:* On the sub-area that is picked according to *Step 2*, the following statistical criteria are further applied to select $v$virtual biopsy samples:

1. Spatial consistency of PI density: For each pixel in the sub-area, place an 8x8 voxel box around it. Then, compute the mean and variance of PI densities over the 64 pixels within the box. Keep the boxes with a low mean (<30%) and a low variance as potential virtual biopsy samples.
2. Separation in the imaging feature space: Good virtual biopsy samples need to be at a certain distance away from each other in the input (imaging features) space – called leverage samples in statistics – in order to stabilize model fitting. To find the leverage samples, we use a highly flexible and efficient clustering algorithm called DBSCAN^3^ to cluster the boxes that have survived sub-step 1) using imaging features. Parameters of DBSCAN are set to produce approximately $v$ clusters. Then, one box from each cluster is picked as the virtual biopsy sample.

**MRI protocol, image co-registration, and normalization**

All imaging was performed on a 3 Tesla system (Sigma HDx; GE-Healthcare, Milwaukee, Wisconsin) within 1 day prior to stereotactic surgery. Conventional MRI included standard pre- and post-contrast T1-Weighted and pre-contrast T2-Weighted sequences. In addition, DTI imaging was performed using Spin-Echo Echo-planar imaging (EPI). We normalized the signal for T1+C, T2W, and EPI+C image datasets using the Simple Insight Segmentation and Registration Toolkit (SimpleITK v1.0.1)^4^ in Python (v3.6.2). The CurvatureFlow algorithm was applied to remove image noise^5^ and the N4ITK algorithm to correct for image intensity nonuniformity bias that could be due to factors such as local magnetic field heterogeneity^6^. Following these corrections, the cerebrospinal fluid (CSF) of the lateral ventricles was used as a reference tissue to normalize the intensity distributions of each dataset using a previously described linear scaling process^7^. Several parametric maps were calculated, such as mean diffusivity (MD) and fractional anisotrophy (FA) based on previously published methods^8^. Also, we acquired DSC-pMRI as previously described and calculated relative cerebral blood volume (rCBV) using IB Neuro^8^. We coregistered multiparametric images from each patient according to ^9^, using tools from ITK (www.itk.org) and IB (Imaging Biometrics) Suite. After coregistration, the imaging data had a plane voxel resolution of ~1.2 mm (256 × 256 matrix) and slice thickness of 3 mm. Following our previous publications^8,10^, we included six multiparametric images in the present study, including T1+C, T2W, EPI+C, MD, FA, and rCBV.


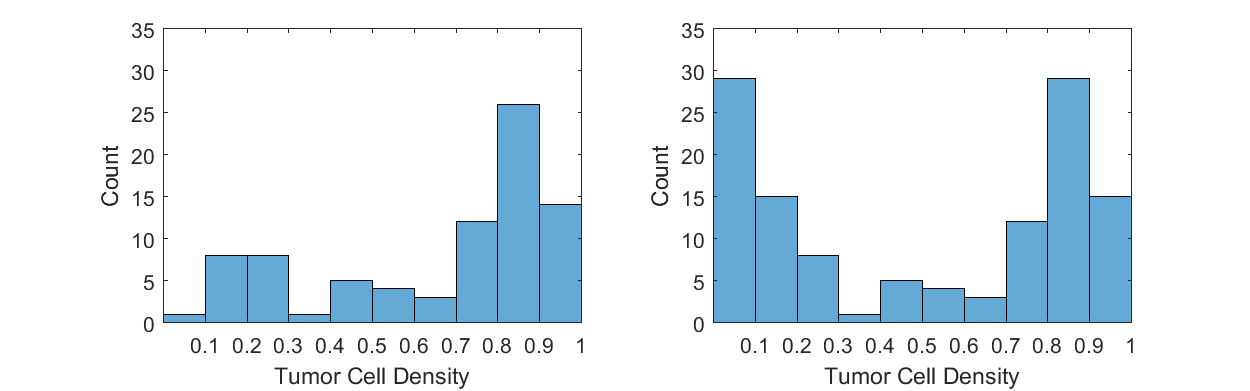


(a) Real biopsies (b) Real and virtual biopsies

**Fig S1. Distribution of cell density in (a) real biopsies, and (b) virtual biopsies.**

**Table S1. Patient-wise MAPEs of ML-PI, PI, and ML**

| Patient | #biopsy samples | ML-PI | PI | ML |
| --- | --- | --- | --- | --- |
| 1 | 7 | **0.086 ± 0.098** | 0.147 ± 0.133 | 0.255 ± 0.163 |
| 2 | 2 | **0 ± 0** | 0.204 ± 0.066 | 0.098 ± 0.034 |
| 3 | 5 | **0.085 ± 0.148** | 0.295 ± 0.158 | 0.229 ± 0.291 |
| 4 | 3 | **0.094 ± 0.039** | 0.157 ± 0.16 | 0.169 ± 0.122 |
| 5 | 2 | **0.006 ± 0.008** | 0.017 ± 0.024 | 0.219 ± 0.289 |
| 6 | 5 | **0.294 ± 0.041** | 0.682 ± 0.067 | 0.508 ± 0.145 |
| 7 | 3 | **0.106 ± 0.184** | 0.172 ± 0.253 | 0.424 ± 0.085 |
| 8 | 6 | **0.117 ± 0.066** | 0.203 ± 0.06 | 0.164 ± 0.121 |
| 9 | 3 | **0.166 ± 0.219** | 0.251 ± 0.337 | 0.24 ± 0.192 |
| 10 | 3 | **0.075 ± 0.09** | 0.144 ± 0.15 | 0.111 ± 0.089 |
| 11 | 6 | **0.044 ± 0.062** | 0.223 ± 0.211 | 0.103 ± 0.096 |
| 12 | 4 | **0.135 ± 0.155** | 0.307 ± 0.304 | 0.229 ± 0.259 |
| 13 | 14 | **0.164 ± 0.166** | 0.243 ± 0.224 | 0.193 ± 0.204 |
| 14 | 4 | 0.119 ± 0.119 | 0.21 ± 0.177 | **0.1 ± 0.124** |
| 15 | 3 | **0 ± 0** | 0.007 ± 0.011 | 0.002 ± 0.004 |
| 16 | 4 | **0.077 ± 0.059** | 0.084 ± 0.101 | 0.22 ± 0.091 |
| 17 | 2 | **0 ± 0** | 0.344 ± 0.224 | 0.158 ± 0.079 |
| 18 | 6 | **0.04 ± 0.043** | 0.169 ± 0.188 | 0.096 ± 0.114 |

**The smallest mean absolute prediction error (MAPE) among the patient specific ML-PI, PI, and ML models is emphasized in bold.**

**Table S2. Patient Specific Tuning Parameters for ML-PI Model**

| Patient | γA | *η* | γI |
| --- | --- | --- | --- |
| 1 | 0.001 | 3 | 1.00E-10 |
| 2 | 0.0001 | 3 | 10000 |
| 3 | 1 | 5 | 10000 |
| 4 | 0.01 | 2 | 1.00E-10 |
| 5 | 1.00E-07 | 70 | 1.00E-07 |
| 6 | 0.01 | 100 | 10000 |
| 7 | 1.00E-07 | 5 | 1000 |
| 8 | 0.0001 | 4 | 100 |
| 9 | 1.00E-06 | 100 | 1.00E-09 |
| 10 | 0.0001 | 50 | 1.00E-10 |
| 11 | 1.00E-06 | 6 | 100 |
| 12 | 1.00E-06 | 4 | 1000 |
| 13 | 0.0001 | 90 | 1.00E-08 |
| 14 | 1.00E-08 | 90 | 100 |
| 15 | 0.001 | 7 | 1.00E-10 |
| 16 | 1.00E-09 | 90 | 1.00E-10 |
| 17 | 1.00E-05 | 9 | 1.00E-10 |
| 18 | 1.00E-05 | 2 | 100 |

**Table S3. Uniform Tuning Parameters for ML-PI Model**

|  | **γA** | **η** | **γI** |
| --- | --- | --- | --- |
| **All Patients** | 1.00E-08 | 100 | 100 |

**Table S4. Partially Uniform Tuning Parameters for ML-PI Model**

| Patient | γA | *η* | γI |
| --- | --- | --- | --- |
| 1 | 1.00E-08 | 5 | 1.00E-07 |
| 2 | 1.00E-09 | 100 | 1000 |
| 3 | 1 | 100 | 10000 |
| 4 | 1.00E-06 | 7 | 1000 |
| 5 | 1.00E-05 | 10 | 1.00E-06 |
| 6 | 0.01 | 100 | 10000 |
| 7 | 1.00E-08 | 4 | 1.00E-05 |
| 8 | 1.00E-07 | 4 | 1000 |
| 9 | 0.0001 | 20 | 1000 |
| 10 | 0.0001 | 50 | 100 |
| 11 | 1.00E-10 | 3 | 10000 |
| 12 | 1.00E-09 | 5 | 1.00E-09 |
| 13 | 0.001 | 90 | 1000 |
| 14 | 1.00E-06 | 9 | 1000 |
| 15 | 1.00E-06 | 80 | 10000 |
| 16 | 1.00E-07 | 40 | 100 |
| 17 | 0.0001 | 6 | 1.00E-10 |
| 18 | 1.00E-06 | 9 | 1000 |

**References**

1. Scholkopf B, Herbrich R, Smola AJ. Generalized Representer Theorem. COLT/EuroCOLT. 2001. p. 416–426.

2. Robnik-Šikonja M, Kononenko I. Theoretical and Empirical Analysis of ReliefF and RReliefF. Mach Learn. 2003; PMID: 1284

3. Ester, M., Kriegel, H. P., Sander, J., & Xu X. A Density-Based Algorithm for Discovering Clusters in Large Spatial Databases with Noise. Kdd. 1996; PMID: 15003161

4. Lowekamp BC, Chen DT, Ibáñez L, Blezek D. The Design of SimpleITK. Front Neuroinform. 2013; PMID: 24416015

5. Sethian JA. Level Set Methods and Fast Marching Methods: Evolving Interfaces in Computational Geometry, Fluid Mechanics, Computer Vision and Materials Science. Cambridge University Press. 1999. PMID: 17220271

6. Tustison NJ, Avants BB, Cook PA, Zheng Y, Egan A, Yushkevich PA, Gee JC. N4ITK: Improved N3 bias correction. IEEE Trans Med Imaging. 2010; PMID: 20378467

7. Mitchell JR, Jones C, Karlik SJ, Kennedy K, Lee DH, Rutt B, Fenster A. MR multispectral analysis of multiple sclerosis lesions. J Magn Reson Imaging. 1997; PMID: 9170034

8. Hu LS, Ning S, Eschbacher JM, Gaw N, Dueck AC, Smith KA, Nakaji P, Plasencia J, Ranjbar S, Price SJ, Tran N, Loftus J, Jenkins R, O’Neill BP, Elmquist W, Baxter LC, Gao F, Frakes D, Karis JP, Zwart C, Swanson KR, Sarkaria J, Wu T, Mitchell JR, Li J. Multi-parametric MRI and texture analysis to visualize spatial histologic heterogeneity and tumor extent in glioblastoma. PLoS One. 2015;10(11). PMCID: PMC4658019

9. Hu LS, Eschbacher JM, Heiserman JE, Dueck AC, Shapiro WR, Liu S, Karis JP, Smith KA, Coons SW, Nakaji P, Spetzler RF, Feuerstein BG, Debbins J, Baxter LC. Reevaluating the imaging definition of tumor progression: Perfusion MRI quantifies recurrent glioblastoma tumor fraction, pseudoprogression, and radiation necrosis to predict survival. Neuro Oncol. 2012; PMID: 22561797

10. Hu LS, Ning S, Eschbacher JM, Baxter LC, Gaw N, Ranjbar S, Plasencia J, Dueck AC, Peng S, Smith KA, Nakaji P, Karis JP, Quarles CC, Wu T, Loftus JC, Jenkins RB, Sicotte H, Kollmeyer TM, O’Neill BP, Elmquist W, Hoxworth JM, Frakes D, Sarkaria J, Swanson KR, Tran NL, Li J, Mitchell JR. Radiogenomics to characterize regional genetic heterogeneity in glioblastoma. Neuro Oncol. 2017;19(1):128–137. PMCID: PMC5193022
